# Negative progesterone receptor status correlates with increased risk of breast cancer recurrence in luminal B HER2-positive and -negative subtypes

**DOI:** 10.20407/fmj.2020-023

**Published:** 2020-12-16

**Authors:** Toshiaki Utsumi, Naomi Kobayashi, Masahiro Hikichi, Kaori Ushimado, Makoto Kuroda

**Affiliations:** 1 Department of Breast Surgery, Fujita Health University, School of Medicine, Okazaki, Aichi, Japan; 2 Department of Breast Surgery, Nagoya Red Cross Hospital, Nagoya, Aichi, Japan; 3 Department of Breast Surgery, Tohoku Kosai Hospital, Sendai, Miyagi, Japan; 4 Department of Diagnostic Pathology, Fujita Health University Okazaki Medical Center, Okazaki, Aichi, Japan

**Keywords:** Breast cancer, Progesterone receptor (PR), Human epidermal growth factor receptor 2 (HER2), Luminal B subtype

## Abstract

**Objectives:**

The prognostic significance of the progesterone receptor (PR) has been widely investigated in luminal A and luminal B [human epidermal growth factor receptor 2 (HER2)–] breast cancer subtypes, both of which are estrogen receptor (ER)-positive and HER2-negative. In contrast, few studies have focused on PR status in luminal B (HER2+) tumors. The aim of this study was to evaluate the impact of positive PR status on outcomes in patients with luminal B (HER2–) or luminal B (HER2+) breast cancer.

**Methods:**

Survival analysis was performed to estimate the likelihood of distant recurrence and death in 469 breast cancer patients with the luminal B (HER2–) or luminal B (HER2+) subtype. The relationship between PR and HER2 status was also assessed.

**Results:**

Of 387 luminal B (HER2–) and 82 luminal B (HER2+) cancers, PR+ was significantly more frequent in the former than the latter (86.3% vs. 61.0%, respectively; *P*<0.001). In univariate analysis, PR was identified as a significant favorable prognostic factor for distant disease–free survival and overall survival in both subtypes, but in multivariate analysis PR was not an independent prognostic factor.

**Conclusions:**

After patients with luminal B subtype were divided into two subgroups according to HER2 status, there was evidence of a relatively good prognosis in the PR+ subgroup. Further studies with a larger number of patients are recommended to validate these findings.

## Introduction

Breast cancer is the most common malignancy in women in many countries.^1^ Recently, microarray analyses and associated technologies have provided new genetic approaches for examining complicated clinical issues regarding breast cancer outcomes.^2,3^ Studies using microarray techniques have revealed that breast cancer is a heterogeneous collection of subtypes distinguished by distinct aberrations at the molecular level. Several studies on gene expression have shown that breast cancer can be classified into at least five specific subtypes: luminal A, luminal B, human epidermal receptor type 2 (HER2)-overexpressing, basal-like, and normal breast-like.^2,3^ Clinical outcomes have been shown to depend on differences in tumor gene expression patterns.^3^

It has been revealed that immunohistochemical methods can serve as a surrogate for multigene microarray analysis to classify breast cancer into subtypes with distinct biological characteristics and clinical outcomes.^4,5^ Therefore, immunohistochemical subtype classification is practical and has been widely used. A statement of the St. Gallen International Expert Consensus includes treatment algorithms based on the classification of breast cancer subtypes according to the immunohistochemistry of estrogen receptor (ER), progesterone receptor (PR), HER2, and Ki67 expression.^6,7^ We previously identified differences in prognosis among breast cancer patients with five distinct immunohistochemical subtypes.^8^

The luminal B subtype is associated with high-grade cancer and poor outcomes.^2^ Interestingly, patients with the luminal B phenotype vary in their prognoses and show different responses to therapeutic strategies. ER and HER2 are important treatment targets in breast cancer.^7,9^ Based on immunohistochemical classification, the luminal B subtype is divided into luminal B (HER2–) and luminal B (HER2+) subtypes according to HER2 status, each of which is associated with different therapeutic strategies.^7,9^ Anti-HER2 therapy is the standard of care for patients with luminal B (HER2+) tumors but not luminal B (HER2–) tumors.^7,9^

The prognostic significance of PR has been widely investigated in both luminal A and luminal B (HER2–) tumors, both of which are ER-positive and HER2-negative.^10–12^ In contrast, few studies have focused on the importance of PR in luminal B (HER2+) tumors.^13,14^ We compared the prognostic significance of PR between luminal B (HER2–) and luminal B (HER2+) breast cancer.

## Patients and Methods

### Subjects

A total of 1,704 patients with breast cancer were treated at Fujita Health University Hospital between January 2003 and December 2014. Patients with stage IV, occult, noninvasive cancer, or bilateral disease were excluded from this study. Male patients with breast cancer and patients lost to follow-up immediately after surgery were also excluded. Among 1,132 women with invasive breast cancer, there were 395 women with luminal B (HER2–) cancer (i.e., HER2–, high Ki67, and either ER+ or PR+) and 85 women with luminal B (HER2+) cancer (HER2+ and either ER+ or PR+). The following women were excluded: eight with luminal B (HER2–) tumors that were ER– and PR+, and three with luminal B (HER2+) tumors that were ER– and PR+. Thus, 387 women with the luminal B (HER2–) subtype and 82 with the luminal B (HER2+) subtype were enrolled in this study.

Histological grade was assessed according to the Bloom and Richardson classification system.^15^ Survival analysis was performed to estimate the likelihood of distant recurrence and death in 469 breast cancer patients with the luminal B (HER2–) or luminal B (HER2+) subtype. We also assessed the relationship between PR status and HER2 status. This retrospective study was approved by the ethics committee of Fujita Health University (No. HM16-138).

### Immunohistochemistry

Immunohistochemical methods were described previously.^8,16,17^ Immunohistochemical staining was performed using the SP1 and 1E2 (Ventana Medical, Tucson, AZ, USA) staining systems for ER and PR, respectively. Positive ER or PR status was defined as the presence of ≥1% positive cancer cells. Immunohistochemical assays for HER2 were performed using the Pathway anti-HER2/neu test (Ventana Medical). Fluorescence in situ hybridization (FISH) was performed using the PathVysion HER-2 DNA probe kit (Abbott France SAS, Rungis, France). An immunohistochemistry score of 3+ or FISH amplification was defined as positive. Ki67 staining was performed using a monoclonal antibody against MIB-1 (Dako, Glostrup, Denmark). The Ki67 labeling index was categorized as low (<14%) or high (≥14%). All markers were assessed with blinding to the clinical data.

### Distant disease-free and overall survival by age group

Distant disease-free survival (DDFS) was calculated from the date of diagnosis to the date of first distant recurrence metastasis or death from any cause. Overall survival (OS) was calculated from the date of diagnosis to the date of death from any cause.^18^ We investigated the prognostic factors for DDFS and OS in univariate analyses, and selected multiple covariates (T stage, pathological node status, PR status, chemotherapy, hormone therapy, and trastuzumab therapy).

### Statistical analysis

Statistical analyses were performed using SPSS 22.0 software (IBM Corp., Armonk, NY, USA). The chi-squared test or Fisher’s exact test was performed for contingency table analysis. Survival curves were generated using the Kaplan–Meier method.^19^ Survival comparisons were made using the log-rank test and Cox proportional hazards multiple regression.

## Results

### Relationship between PR status and HER2 status

Luminal B cancers were subclassified into luminal B (HER2–) and luminal B (HER2+) cancers according to HER2 status, then further subclassified into luminal B (HER2–) PR+ or luminal B (HER2+) PR+ cancers if PR was positive, and luminal B (HER2–) PR– or luminal B (HER2+) PR– cancers if PR was negative. Of the 469 cancers, 82.5% (n=387) were luminal B (HER2–) and 17.5% (n=82) were luminal B (HER2+). Of the 387 luminal B (HER2–) cancers, 86.3% were PR+ and 13.7% were PR–, and of the 82 luminal B (HER2+) cancers, 61.0% were PR+ and 39.0% were PR–. Luminal B (HER2–) cancers were PR+ significantly more common than luminal B (HER2+) cancers (86.3% vs. 61.0%, respectively; *P*<0.001, Table 1).

### Clinical characteristics of luminal B (HER2–) and luminal B (HER2+) cancers by PR status

Table 2 shows the clinical profiles of the 469 patients with luminal B (HER2–) or luminal B (HER2+) cancer. The patients’ median age was 55 years (range, 22–90). Luminal B (HER2+) PR+ cancers were significantly more likely to be early-stage (stage I) than luminal B (HER2+) PR– cancers (32.0% vs. 15.6%, respectively; *P*=0.001).

Data on pathologic node status were missing for 11 women: eight with luminal B (HER2–) cancer and three with luminal B (HER2+) cancer. Of the eight patients with luminal B (HER2–) cancer, seven did not undergo axillary surgery. The remaining patient and the three patients with luminal B (HER2+) cancer had no pathological node involvement after neoadjuvant chemotherapy and no evidence of negative lymph node status before neoadjuvant chemotherapy.

No data were available on grade in six patients with luminal B (HER2–) cancer and four patients with luminal B (HER2+) cancer. Lesions that were luminal B (HER2+) PR+ were less likely to be of unknown histological grade than those that were luminal B (HER2+) PR– (0% vs. 12.5%, respectively; *P*=0.05).

Regarding the relationship between medical treatment and subtypes, chemotherapy was used in 94.0% of patients with luminal B (HER2+) PR+ cancer and 68.8% of patients with luminal B (HER2+) PR– cancer (*P*=0.003) (Table 2).

Next, we investigated the relationship between surgical treatment and the four cancer subtypes. The rate of breast-conserving surgery in patients with luminal B (HER2+) PR+ cancer was significantly higher than that in patients with luminal B (HER2+) PR– cancer (*P*=0.028).

### DDFS and OS by PR status

**Figure 1:**
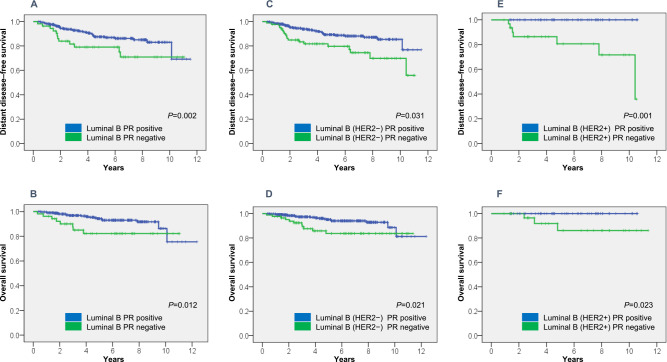
DDFS and OS in 469 women with breast cancer. (A) DDFS in patients with luminal B cancer, (B) OS in patients with luminal B cancer, (C) DDFS in patients with luminal B (HER2–) cancer, (D) OS in patients with luminal B (HER2–) cancer, (E) DDFS in patients with luminal B (HER2+) cancer, and (D) OS in patients with luminal B (HER2+) cancer.

With an overall median follow-up of 4.59 [4.88 (range, 0.40–12.37) years for women with PR+ cancer and 4.47 (range, 0.32–11.37) years for those with PR– cancer], the estimated 5-year DDFS rate was 89.3±1.8% for luminal B PR+ and 79.7±4.8% for luminal B PR– (*P*=0.002) (Table 3 and Figure 1A). The estimated 5-year OS rate was 95.1±1.3% for luminal B PR+ and 83.7±4.6% for luminal B PR– (*P*=0.012) (Table 3 and Figure 1B).

The estimated 5-year DDFS rate was 87.5±2.1% for luminal B (HER2–) PR+ and 79.0±5.9% for luminal B (HER2–) PR– (*P*=0.031) (Table 3 and Figure 1C), while the estimated 5-year OS rate was 94.3±1.5% for luminal B (HER2+) PR+ and 82.2±5.8% for luminal B (HER2+) PR– (*P*=0.021) (Table 3 and Figure 1D). The estimated 5-year DDFS rate was 100% for luminal B (HER2+) PR+ and 80.6±8.1% for luminal B (HER2+) PR– (*P*=0.001) (Table 3 and Figure 1E). The estimated 5-year OS rate was 100% for luminal B (HER2+) PR+ and 86.1±7.6% for luminal B (HER2+) PR– (*P*=0.023) (Table 3 and Figure 1F).

Patients with PR+ disease generally had more favorable outcomes than those with PR– disease.

### Multivariate survival analysis

In women with luminal B (HER2–) cancer, univariate analyses showed that for DDFS, T stage and node status were significant unfavorable prognostic factors and PR was a significant favorable prognostic factor, while for OS, T stage and node status were significant unfavorable prognostic factors and PR and hormone therapy were significant favorable prognostic factors. PR and trastuzumab therapy were significant favorable prognostic factors for both DDFS and OS in women with luminal B (HER2+) cancer.

Chemotherapy was not associated with DDFS or OS. A multivariate analysis to determine independent predictors of survival in women with luminal B (HER2–) cancer identified node status as significant for DDFS, and node status and hormone therapy as significant for OS (Table 4). Trastuzumab therapy was a significant prognostic factor for DDFS in luminal B (HER2+) tumors (Table 4).

## Discussion

Several studies have investigated the importance of PR as a prognostic factor in luminal A and luminal B (HER2–) breast cancer subtypes, but few have evaluated the relationship between PR status and the luminal B (HER2+) subtype.^10–14^ Since the PR is the end product of estrogen action, PR expression is generally thought to depend on ER activity, with a lack of PR reflecting a nonfunctional ER and resistance to hormonal therapy.^20,21^ Some studies have identified alternative molecular mechanisms that may explain the loss of PR.^22,23^ Experimental results have suggested that differences in outcomes and selective ER modulator resistance among ER+/PR– breast cancers are because of growth factors that reduce PR levels.^22,23^

In this study, we examined the relationship between PR and HER2 status, the clinical characteristics of the luminal B (HER2–) and luminal B (HER2+) subtypes, and the prognostic importance of PR in these two subtypes.

We found an inverse correlation between PR and HER2 status, which is consistent with the results of previous studies.^23–26^ Some studies have shown that ER+ and PR– tumors express higher levels of HER1 and HER2 and display more aggressive features than ER+ and PR+ tumors.^22^ The HER family lies upstream of the phosphatidylinositol 3-kinase (PI3K)/serine-threonine protein kinase (AKT)/mammalian target of rapamycin (mTOR) signaling pathway.^27^ Moreover, a recent preclinical study demonstrated that PR expression was reduced via the PI-3K/AKT pathway.^28^ These findings may support our clinical results.

Luminal B (HER2+) PR+ cancers were significantly more likely to be early-stage (stage I) than luminal B (HER2+) PR– cancers (32.0% vs. 15.6%, respectively; *P*=0.001), but there was no significant difference in stage between the luminal B (HER2–) PR+ and luminal B (HER2–) PR– subtypes. We have insufficient data to explain the reasons for these results. The luminal B (HER2+) PR+ subgroup received chemotherapy more frequently than the luminal B (HER2+) PR– subgroup, probably because the proportion of patients over 70 years old was almost twice as high in the latter subgroup than in the former.

Interestingly, univariate analysis showed that PR was a significant prognostic factor for DDFS and OS in both the luminal B (HER2–) and luminal B (HER2+) subtypes. These findings are similar to those identified in a study by Cancello et al.^13^ Multivariate analysis, however, showed that PR was not an independent predictor of survival in either subtype; possible reasons include the fact that PR is a relatively weak prognostic factor and the sample size was small. If we can identify a high-risk group in the luminal B (HER2–) and/or luminal B (HER2+) subtypes, we should consider a strategy for personalized therapy for improving the outcomes of this high-risk group. For example, if the prognosis of patients with PR-negative tumors in these subtypes is definitely poor, we should consider increasing the intensity of the treatment for these patients to improve their prognosis.

Our study has several limitations. First, it was a retrospective, single-center study and therefore may have been prone to selection bias. Second, the number of patients was relatively small, especially in the group with the luminal B (HER2+) subtype. Since small studies cannot yield definitive results, care should be taken when interpreting our findings. A larger observational series might yield additional data. Despite these limitations, our study has several strengths. First, it analyzed precise data regarding pathological factors and clinical outcomes in both subtype groups. Second, the significance of PR status was well characterized in the luminal B (HER2–) subtype, but not in the luminal B (HER2+) subtype. Our study indicates the need for research on the biological significance of PR in the latter subtype.

In conclusion, after dividing the luminal B subtype group into two subgroups according to HER2 status, we provided evidence of a relatively good prognosis in the PR+ subgroup. Further studies with a larger number of patients are recommended to validate our findings.

## Figures and Tables

**Table1:** Relationship between PR and HER2 in 469 women with luminal B breast cancer

	Luminal B (HER2–)	Luminal B (HER2+)	*P*-value
PR+	334 (86.3%)	50 (61.0%)	
PR–	53 (13.7%)	32 (39.0%)	<0.001

PR, progesterone receptor; HER2, human epidermal growth factor receptor 2

**Table2:** Clinical profiles of the 469 patients

	Luminal B (HER2–)				Luminal B (HER2+)		
	
	PR+	PR–	*P*-value		PR+	PR–	*P*-value
Number of patients	334	53			50	32	
Age (years)				
≤39	46 (13.8%)	10 (18.9%)			8 (16.0%)	2 (6.3%)	
40–49	87 (26.0%)	8 (15.1%)			22 (44.0%)	7 (21.9%)	
50–59	71 (21.3%)	7 (13.2%)			9 (18.0%)	10 (31.3%)	
60–69	75 (22.5%)	17 (32.1%)			6 (12.0%)	6 (18.8%)	
≥70	55 (16.5%)	11 (20.8%)	0.147		5 (10.0%)	7 (21.9%)	0.085
Stage				
I	121 (36.2%)	12 (22.6%)			16 (32.0%)	5 (15.6%)	
IIA	133 (39.8%)	20 (37.7%)			16 (32.0%)	12 (37.5%)	
IIB	53 (15.9%)	12 (22.6%)			17 (34.0%)	4 (12.5%)	
IIIA	11 (3.3%)	3 (5.7%)			0 (0%)	6 (18.8%)	
IIIB	13 (3.9%)	5 (9.4%)			1 (2.0%)	4 (12.5%)	
IIIC	3 (0.9%)	1 (1.9%)	0.161		0 (0%)	1 (3.1%)	0.001
T stage				
T1	132 (39.5%)	14 (26.4%)			16 (32.0%)	8 (25.0%)	
T2–4	202 (60.5%)	39 (73.6%)	0.067		34 (68.0%)	24 (75.0%)	0.497
Pathological node status				
Negative	189 (56.6%)	25 (47.2%)			24 (48.0%)	11 (34.4%)	
Positive	137 (41.0%)	28 (52.8%)			24 (48.0%)	20 (62.5%)	
Unknown	8 (2.4%)	0 (0%)	0.174		2 (4.0%)	1 (3.1%)	0.437
Histological grade				
1	48 (14.4%)	3 (5.7%)			6 (12.0%)	6 (18.8%)	
2	186 (55.7%)	29 (54.7%)			37 (74.0%)	18 (56.3%)	
3	94 (28.1%)	21 (39.6%)			7 (14.0%)	4 (12.5%)	
Unknown	6 (1.8%)	0 (0%)	0.132		0 (0%)	4 (12.5%)	0.05
Chemotherapy				
Given	169 (50.6%)	33 (62.3%)			47 (94.0%)	22 (68.8%)	
Not given	165 (49.4%)	20 (37.7%)	0.114		3 (6.0%)	10 (31.3%)	0.003*
Endocrine therapy				
Given	274 (82.0%)	41 (77.4%)			48 (96.0%)	27 (84.4%)	
Not given	60 (18.0%)	12 (22.6%)	0.416		2 (4.0%)	5 (15.6%)	0.078*
Trastuzumab				
Given	3 (0.9%)	0 (0%)			38 (76.0%)	22 (68.8%)	
Not given	331 (99.1%)	53 (100%)	0.489		12 (24.0%)	10 (31.3%)	0.470
Breast surgery				
BCS	197 (59.0%)	30 (56.6%)			28 (56.0%)	10 (31.3%)	
Mastectomy	137 (41.0%)	23 (43.3%)	0.744		22 (44.0%)	22 (68.8%)	0.028
Axillary surgery				
No surgery	7 (2.1%)	0 (0%)			0 (0%)	0 (0%)	
ALND±SNB	127 (38.0%)	26 (49.1%)			29 (58.0%)	21 (65.6%)	
SNB	200 (59.9%)	27 (50.9%)	0.208		21 (42.0%)	11 (34.4%)	0.490

* Fisher’s exact test ER, estrogen receptor; PR, progesterone receptor; HER2, human epidermal growth factor receptor 2; BCS, breast-conserving surgery; ALND, axillary lymph node dissection; SNB, sentinel lymph node biopsy

**Table3:** DDFS and OS by PR status

	Estimated 5-years DDFS	*P*-value	Estimated 5-years OS	*P*-value
All patients				
PR+	89.3±1.8%		95.1±1.3%	
PR–	79.7±4.8%	0.002	83.7±4.6%	0.012
Luminal B (HER2–) subgroup				
PR+	87.5±2.1%		94.3±1.5%	
PR–	79.0±5.9%	0.031	82.2±5.8%	0.021
Luminal B (HER2+) subgroup				
PR+	100%		100%	
PR–	80.6±8.1%	0.001	86.1±7.6%	0.023

DDFS, distant disease-free survival; OS, overall survival; PR, progesterone receptor; HER2, human epidermal growth factor receptor 2

**Table4:** Multivariate Cox analysis of DDFS and OS

Covariate	DDFS					OS			
	
	Univariate		Multivariate			Univariate		Multivariate	
			
	*P* value		*P*-value	Hazard ratio (95% CI)		*P*-value		*P*-value	Hazard ratio (95% CI)
Luminal B (HER2–) subgroup									
T stage (T2–T4/T1)	0.014		0.137			0.047		0.093	
Node status (positive/negative)	<0.001		<0.001	6.112 (2.827–13.214)		0.001		0.004	4.114 (1.591–10.636)
PR (positive/negative)	0.031		0.151			0.021		0.360	
Hormone therapy (yes/no)						0.008		0.003	0.266 (0.112–0.632)
Luminal B (HER2+) subgroup									
PR (positive/negative)	0.001		0.944			0.023		0.385	
Trastuzumab therapy (yes/no)	0.005		0.037	0.101 (0.012–0.871)		0.009		0.372	

DDFS, distant disease-free survival; OS, overall survival
